# Editorial: Creativity and Mental Imagery

**DOI:** 10.3389/fpsyg.2016.01280

**Published:** 2016-08-25

**Authors:** Massimiliano Palmiero, Laura Piccardi, Raffaella Nori, Liana Palermo, Carola Salvi, Cecilia Guariglia

**Affiliations:** ^1^Neuropsychology Unit, Fondazione Santa Lucia, I.R.C.C.SRome, Italy; ^2^Department of Life, Health and Environmental Sciences, University of L'AquilaL'Aquila, Italy; ^3^Department of Psychology, University of BolognaBologna, Italy; ^4^Department of Medical and Surgical Sciences, Magna Græcia University of CatanzaroCatanzaro, Italy; ^5^Department of Psychology, Northwestern UniversityEvaston, IL, USA; ^6^Rehabilitation Institute of ChicagoChicago, IL, USA; ^7^Department of Psychology, Sapienza University of RomeRome, Italy

**Keywords:** creativity, divergent thinking, convergent thinking, insight, imagery, attention

Considering the pivotal role that creative ideas play in human societies, and creativity's contribution to multiple aspects of human life, understanding the cognitive components underlying creativity has become increasingly fundamental. Since the Five-Stages Model of the creative process proposed by Wallas (1926), creativity has become associated with topics as wide-ranging as from problem-solving (Plucker et al., 2004) to art (van Leeuwen et al., 1999; Batt et al., 2010). Furthermore, creativity has been identified as a predictor for educational success and wellbeing (Plucker et al., 2004), and has been proposed as a way to improve the quality of life in healthy and pathological aging (Cohen, 2006; Palmiero et al., 2012, 2014, 2016a,b; Palmiero, 2015).

In the present Frontiers in Cognition Research Topic 11 novel publications were collected: 8 Original Research Articles, 1 Review, and 2 Perspective Articles. From the beginning, the Research Topic was planned as a collection of studies exploring the relationships between creativity and mental imagery. Mental imagery is a representational medium for providing researchers access to thoughts, symbolization, and combination of elements, possibly facilitating the emergence of new ideas and creativity. In this direction, different aspects of mental imagery were considered which could increase or explain the emergence of creativity: daydreaming styles (common forms of imagination that involve spontaneous thoughts unrelated to the context, Zedelius and Schooler); imagination of activities over a long period of time, relevant especially for actual creative achievements in science and writing (Jung et al.); as well as ‘looking at nothing’ and blinking behaviors, that do not necessarily involve visual imagery (Salvi and Bowden). In addition, we explored the relationships between different creative objects' production and artistic drawings with different mental imaging processes (i.e., generation, inspection and transformation, Palmiero et al.).

We also collected studies that investigated distinct and peculiar aspects of creativity and its cognitive components, such as: the equal-odds rule of divergent thinking, also known as the relationships between fluency (the number of responses) and creativity as assessed by independent judges (Jung et al.); or the relationships between flexibility of divergent thinking (the number of categories encompassing the relevant responses) and attentive processing (Zmigrod et al.). Interestingly, the relationships between convergent thinking involving insight and intelligence (Zmigrod et al.), and working memory updating (that is maintenance of items in working memory and binding of the incoming information, Necka et al.). In addition, neural correlates of creativity were investigated. Chavez highlighted the key role of brain areas involved in motor imagery on highly creative individuals, whereas Boccia et al. showed that general creativity relies on multi-componential neural networks supporting executive functions, whereas domain-specific creativity (verbal, musical and visuo-spatial) roughly depends on different functional specialized brain regions.

Finally, two different tests recently developed have been reported: the Test of Creative Imagery Abilities (Jankowska and Karwowski), aimed at assessing three components of creative imagination: vividness of imagery, originality of responses, and transformative imagery ability; and the Artistic Creativity Domains Compendium (Lunke and Meier), aimed at measuring artistic creativity in visual arts, performing arts, literature and music.

Taken together, the articles included in this Research Topic bring up novel perspectives for better understanding creativity as a cognitive process and its relation with mental imagery. Despite, the role of mental imagery in creativity has been robustly supported, several issues remains to be addressed to clarify the extent to which different forms, abilities and strategies of imagery affect creative idea generation, for example, the subcomponents of the relationships between imagery and creativity in specific domains of knowledge. Apart from imagery, the Research Topic also highlights the key role of attention in creativity, opening up the question of how attention might increase creativity in different ways. Finally, the neural bases of creativity need to be further investigated since there is no agreement about the brain areas specialized for creativity.

In conclusion, the variety of approaches and methods to measure creativity and its components makes difficult to draw clear conclusion about this topic. In future studies, comparing special groups of subjects in normal and pathological conditions (e.g., artists, designers, mathematicians, patients with dementia, brain-damaged patients and so forth) might help to better understand the cognitive and neural correlates of creativity and the relationships among creativity and other cognitive domains, such as mental imagery, attention, and problem solving. We hope that the papers included in this Research Topic can help to stimulate more studies on these topics and in increasing research in the field of creativity.

## Author contributions

MP: Planned the topic and edited the most of papers included in the topic. LaP: Edited some papers included in the topic. RN: Edited some papers included in the topic. LiP: Edited some papers included in the topic. CS: Edited some papers included in the topic. CG: Edited some papers included in the topic.

## Funding

This work was supported by NIH [grant number T32 NS047987] to CS.

### Conflict of interest statement

The authors declare that the research was conducted in the absence of any commercial or financial relationships that could be construed as a potential conflict of interest.
